# Ginsenoside Rg1 Regulates SIRT1 to Ameliorate Sepsis-Induced Lung Inflammation and Injury via Inhibiting Endoplasmic Reticulum Stress and Inflammation

**DOI:** 10.1155/2019/6453296

**Published:** 2019-02-24

**Authors:** Qian-Lu Wang, Lei Yang, Yue Peng, Min Gao, Ming-Shi Yang, Wei Xing, Xian-Zhong Xiao

**Affiliations:** ^1^Department of Intensive Care Medicine, The Third Xiangya Hospital of Central South University, Changsha 410013, China; ^2^Department of Pharmacy, The First Hospital of Hunan University of Chinese Medicine, Changsha 410003, China; ^3^Department of Pathophysiology, Xiangya School of Medicine, Central South University, Changsha 410078, China

## Abstract

**Objectives:**

To investigate the protective effect of ginsenoside Rg1 on relieving sepsis-induced lung inflammation and injury *in vivo* and *in vitro*.

**Methods:**

Cultured human pulmonary epithelial cell line A549 was challenged with LPS to induce cell injury, and CLP mouse model was generated to mimic clinical condition of systemic sepsis. Rg1 was applied to cells or animals at indicated dosage. Apoptosis of cultured cells was quantified by flow cytometry, along with ELISA for inflammatory cytokines in supernatant. For septic mice, lung tissue pathology was examined, plus ELISA assay for serum cytokines. Western blotting was used to examine the activation of inflammatory pathways and ER stress marker proteins in both cells and mouse lung tissues. Reactive oxygen species (ROS) level was quantified by DCFDA kit.

**Results:**

Ginsenoside Rg1 treatment remarkably suppressed apoptosis rate of LPS-induced A549 cells, relieved mouse lung tissue damage, and elevated survival rate. Rg1 treatment also rescued cells from LPS-induced intracellular ROS. In both A549 cells and mouse lung tissues, further study showed that Rg1 perfusion significantly suppressed the secretion of inflammatory cytokines including tumor necrosis factor- (TNF-) alpha and interleukin- (IL-) 6 and relieved cells from ER stress as supported by decreased expression of marker proteins via upregulating sirtuin 1 (SIRT1).

**Conclusion:**

Our results showed that ginsenoside Rg1 treatment effectively relieved sepsis-induced lung injury *in vitro* and *in vivo*, mainly via upregulating SIRT1 to relieve ER stress and inflammation. These findings provide new insights for unrevealing potential candidate for severe sepsis accompanied with lung injury.

## 1. Introduction

Sepsis is a severe systemic condition secondary to inflammatory outbreak. Although life support system is continuously evolving, the prognosis of sepsis, especially septic shock, is still unfavorable. Current treatment approach mainly targets the infectious pathogens using wide-spectrum antibiotics but largely ignores cellular damage caused by sepsis, which can lead to severe complications for mortality. Endoplasmic reticulum (ER) stress is an important cellular event in septic patients [1]. The investigation of ER stress mechanism in sepsis thus can benefit potential treatment strategy development. Ginsenoside Rg1 is one of the active compounds extracted from Chinese herb ginseng and has shown promising effects in various pathological conditions. Previous study has shown that ginsenoside Rg1 could protect cells from ER stress under various conditions such as nephropathy [2], diabetic cardiomyopathy [3], or Alzheimer's disease [4]. We thus expect that Rg1 might exert similar roles in alleviating ER stress under septic challenge. However, no study has been made regarding the role of Rg1 in protecting sepsis-related lung injury by alleviating ER stress.

Sirtuin 1 (SIRT1) is a NAD^+^-dependent deacetylase and has been reported to modulate various protein functions. The protective role of SIRT1 has been proven in multiple pathological conditions including atherosclerosis [5], diabetic cardiomyopathy [6], and cardiomyopathy [7]. Generally, protective effects of SIRT1 are shown to be correlated with alleviation of ER stress, such as those in diabetic cardiomyopathy [6]. Moreover, a recent publication demonstrated that SIRT1 could reverse sepsis-induced myocardial injury via anti-inflammation and relieving ER stress [8]. Another study reported that miR-199a aggravates LPS-induced inflammatory response via targeting SIRT1 in acute respiratory distress syndrome (ARDS) [9]. Therefore, we hypothesized that ginsenoside Rg1 might ameliorate sepsis-induced cell ER stress and apoptosis via upregulating SIRT1, eventually protecting against lung inflammation or injury.

In present study, we thus performed both *in vitro* and *in vivo* studies to examine the effect of ginsenoside Rg1 on relieving sepsis-induced injury. Using both cultured human lung epithelial cell line A549 and septic mouse models, we showed that Rg1 treatment effectively alleviated cell death and tissue injury, which can be largely attributed to suppressed ER stress plus lower secretion of inflammatory cytokines via upregulating SIRT1. Our results provide more evidences for using Rg1 as the potential drug candidate for treating sepsis-related lung injury.

## 2. Materials and Methods

### 2.1. Cell Culture and LPS Induction

Human pulmonary epithelial cell line A549 was purchased from American Type Culture Collection (ATCC) and was cultured in DMEM medium (Gibco, US) containing 10% fetal bovine serum, 100 U/ml penicillin, and 100 ug/ml streptomycin. Cells were grown on culture dishes until achieving 80%~90% confluence. To mimic *in vitro* sepsis model, LPS (1 *μ*g/ml) purchased from Sigma-Aldrich (USA) was added into a culture medium for 24 h. Rg1 purchased from E-research Biological Technology (Shanghai, China) was added at indicated end concentrations into a culture medium 30 min after LPS challenge.

### 2.2. Animal Model

To generate a mouse sepsis model, CLP (cecal ligation and puncture) approach was applied as previously reported [10]. In brief, C57BL/6J mice (7~8 weeks old, *n* = 10 for the sham group, *n* = 20 for the other groups, provided by Laboratory Animal Center of Central South University) were housed in specific pathogen-free environment. Under general anesthesia, a midline laparotomy was performed to expose the cecum, which was ligated to the ileocecal valve. The distal part of the cecum was punctured using a syringe needle. Cecum content was purposely squeezed into the abdominal cavity for inducing systemic sepsis. 1 ml PBS was used for fluid resuscitation before closing the abdomen. Rg1 (10 mg/kg or 20 mg/kg) was intravenously infused 30 min after surgery. All CLP mice presented severe sepsis symptoms within 24 h and died mostly within 48 h. Animal protocol has been reviewed and approved by the ethical committee of Central South University. (Approval no. LLSC (LA) 2017-060). Animal experiments were repeated three times. Tissues from all possible animals were prepared for the detection of indicated index. For statistical analysis, data from three animals in each animal experiment were averaged and three mean values from three experiments were finally averaged.

### 2.3. Flow Cytometry for Cell Apoptosis

A549 cells were seeded into a culture plate at 2 × 10^6^ density. After 24 h incubation, cells were quantified using annexin V-FITC apoptotic kit (Beyotime, Jiangsu, China) as previously described [11]. Briefly speaking, cells were harvested and washed in PBS for two times and were resuspended in annexin V binding buffer. FITC-conjugated annexin V and propidium iodide (PI) was added successively. With 10 min dark incubation at room temperature, cell mixture was loaded onto flow cytometry system (CytoFLEX, Beckman Coulter) for quantification of apoptotic cells.

### 2.4. ELISA for Inflammatory Cytokines

The levels of inflammatory cytokines including interleukin- (IL-) 1*β*, tumor necrosis factor- (TNF-) *α*, and interleukin- (IL-) 6 were measured in both cultured A549 cells and serum from CLP mice. In brief, cultured A549 cells were treated with LPS for different time points and were treated with ginsenoside Rg1 at gradient concentrations. The supernatant of cultured cells was extracted for quantifying the concentration of IL-1*β*, TNF-*α*, and IL-6 using ELISA kits (Boster Biological Technology, Wuhan, China) following manual instructions. For CLP mice, blood samples were collected from the tail vein and were centrifuged to collect the serum. TNF-*α* and IL-6 levels were then determined using ELISA kits.

### 2.5. Western Blotting

Western blotting was used to quantify the expression levels of inflammatory markers (p-p65, iNOS, 1 : 1000, Cell Signaling Technology), SIRT1 and ER stress markers (CHOP, GRP78, IRE1*α*, and ATF6, 1 : 1000, Cell Signaling Technology) in both A549 cells and CLP mouse lung tissues. In brief, cells or tissues were lysed in RIPA buffer and total protein concertation was quantified by BCA kit (Beyotime, Jiangsu, China). About 20~40 *μ*g protein samples (equalized based on BCA determined total protein concentration) were loaded onto SDS-PAGE gel for electrophoresis separation and were transferred to PVDF membrane. After blocking, the membrane was incubated with primary antibody including p-p65 (#3065), p65 (#8242), iNOS (#13120), IRE1*α* (#3294), CHOP (#2895), GRP78 (#3177), ATF6 (#65880), SIRT1 (#8469), and *β*-actin (#4970, all in 1 : 1500 dilution, from Cell Signaling Technology) overnight. On the next day, HRP-conjugated secondary antibody was added for room temperature incubation. The membrane was then developed by ECL substrate, and images were captured by a computerized system (ChemiScope 6000, Clinx, Shanghai, China). Quantification was performed using ImageJ software, and statistical analysis was done based on at least 3 mean values from three independent experiments. Relative protein level of each sample was determined as gray level of individual protein divided by the respective gray level of *β*-actin.

### 2.6. ROS Assay

Intracellular level of reactive oxygen species (ROS) was measured for reflecting ER stress level of cultured cells, using a DCFDA cellular ROS detection assay kit (Abcam, US) following the manual instruction. ROS level was determined by measuring the fluorescent intensity of DCFDA at 535 nm using fluorescent-activated cell sorting (FACS) approach, on a FACSCanto II™ cytometer (BD, US).

### 2.7. HE Staining

CLP mice were sacrificed and lung tissues were extracted. Tissues were fixed in paraformaldehyde, embedded into paraffin, and were sectioned into 4~5 *μ*m slices. Tissue sections were firstly dewaxed in xylene and were rehydrated in graded ethanol. Sections were then sequentially immersed in hematoxylin for 10 min and in eosin for 1 min. Then tissues were rinsed in distilled water and were dehydrated for coverslip mounting. Images were taken under a light field microscope for analysis. Lung injury was evaluated and scored by two pathologists blinded to the experimental setup using a recent criterion. Briefly, 0 to 4 (none, light, moderate, severe, and very severe) for the following categories: neutrophil infiltration, pulmonary edema, and disorganization of lung parenchyma and hemorrhage, was scored. A sum injury score was calculated by adding individual scores in every animal in each group and averaged.

### 2.8. Statistical Analysis

All data were presented as mean ± standard deviation (SD). Parametric data were compared by student *t*-test, and one-way ANOVA was used for multiple comparisons followed by Tukey post hoc test. *P* < 0.05 was considered to be statistically significant. All statistical analysis was performed using SPSS18.0 software.

## 3. Results

### 3.1. Ginsenoside Rg1 Inhibits Cell Apoptosis and Relieves ROS Generation in LPS-Induced A549 Cells

**Figure 1:**
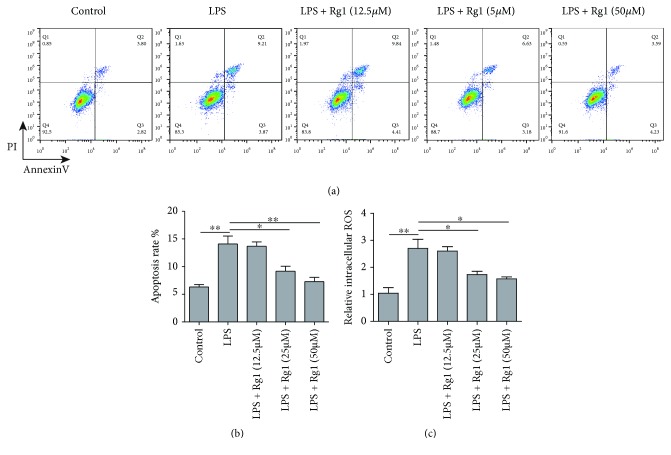
Rg1 attenuates LPS-induced A549 cell apoptosis and ROS. (a) Flow cytometry results for apoptosis rate of cultured A549 cells using annexin V/PI double labelling approach. (b) Quantification of apoptotic rate with LPS induction and gradient Rg1 treatment. (c) Relative levels of intracellular reactive oxygen species (ROS) in A549 cells. ^∗^*P* < 0.05, ^∗∗^*P* < 0.01, compared to the control or LPS model group. Data were shown as mean + SD based on three independent experiments.

We firstly investigated the effect of ginsenoside Rg1 on protecting lung tissues from sepsis-induced cell apoptosis and intracellular ROS spike using A549 epithelial cells as an *in vitro* model. Using flow cytometry and annexin V-FITC/PI dual staining approach, we found that LPS treatment remarkably elevated the apoptotic rate, and treatment of Rg1 at 25 and 50 *μ*M decreased cell apoptosis, with more potent effect in the 50 *μ*M group (Figures 1(a) and 1(b)). We further quantified intracellular ROS level using fluorescent probe method. In consistent with apoptotic assay, LPS remarkably increased the intracellular ROS level, which was suppressed by Rg1 at 25 and 50 *μ*M treatment (Figure 1(c)). All these data thus suggested that ginsenoside Rg1 could protect cells from LPS-induced ROS to prevent cell death.

### 3.2. Rg1 Suppressed Release of Inflammatory Cytokines from LPS-Induced A549 Cells

**Figure 2:**
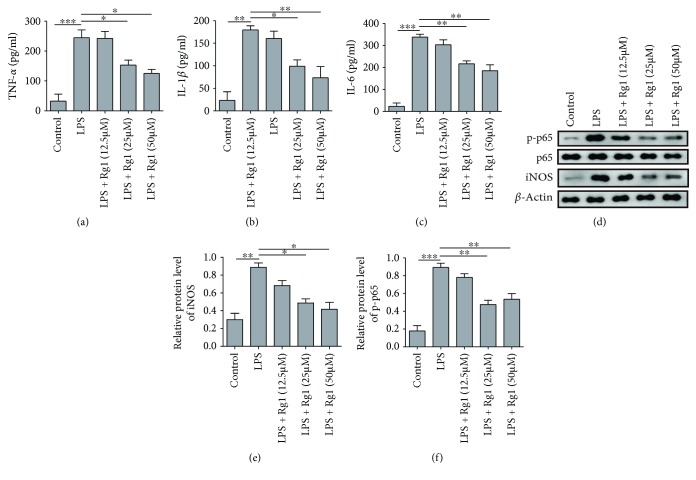
Rg1 attenuates inflammation response in LPS-induced A549 cell. (a–c) Secreted level of TNF-*α*, IL-1*β*, and IL-6 in cultured A549 cells. (d) Representative images for the expression of p-p65 and iNOS proteins in cultured cells after Rg1 treatment. (e, f) Quantification of relative expression level of iNOS and p-p65. ^∗^*P* < 0.05; ^∗∗^*P* < 0.01; ^∗∗∗^*P* < 0.01, compared to the control or LPS group. Data were shown as mean + SD based on three independent experiments.

To further investigate the mechanism of protective effects of ginsenoside Rg1 on lung epithelial cells, we quantified the release of inflammatory cytokines from cultured A549 cells. ELISA assay showed that LPS induction strongly activated secretion of inflammatory cytokines including TNF-*α*, IL-1*β*, and IL-6. Treatment using Rg1 at 25 and 50 *μ*M, but not 12.5 *μ*M, remarkably suppressed secretion of inflammatory cytokines (Figures 2(a)–2(c)). Therefore, Rg1 also suppressed the secretion of inflammatory cytokines from lung cells besides antiapoptotic effects. We also examined the upstream mediator for inflammatory cytokines including p-p65 (marker for activation of NF-*κ*B) and downstream target iNOS (inducible nitric oxide synthase). Consistently, Western blotting results showed that LPS induction elevated iNOS and p-p65 expression, whilst Rg1 treatment suppressed expression of these two proteins (Figures 2(d)–2(g)). Collectively, these data showed that Rg1 can protect cells from LPS-induced inflammatory response in A549.

### 3.3. Rg1 Relieved ER Stress in LPS-Induced A549 Cells

**Figure 3:**
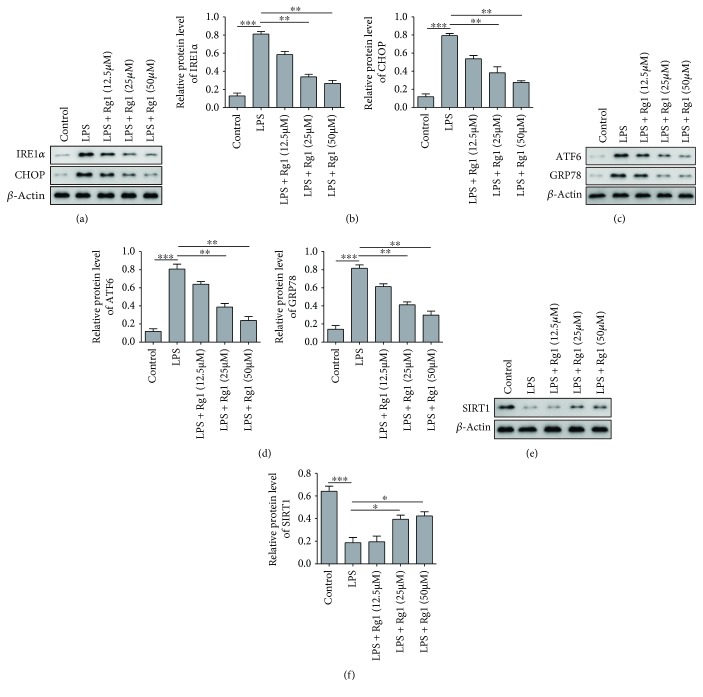
Rg1 attenuates ER stress in LPS-induced A549 cell. (a) Representative images for the expression of IRE1*α* and CHOP proteins in Rg1-treated cells by Western blotting. (b) Quantification of relative expression of IRE1*α* and CHOP proteins. (c) Representative images for ATF6 and GRP78 expression by Western blotting. (d) Quantification of relative expression levels of ATF6 and GRP78. (e) Representative images for SIRT1 protein expression by Western blotting. (f) Quantification of relative expression level of SIRT1. ^∗^*P* < 0.05; ^∗∗^*P* < 0.01; ^∗∗∗^*P* < 0.01, compared to the control or LPS model group. Data were shown as mean + SD based on three independent experiments.

Next, we aimed to examine whether these protective effects of Rg1 on LPS-treated A549 cells were related with ER stress. Western blotting showed that LPS treatment significantly elevated the expression of ER stress marker proteins including CHOP, GRP78, IRE1*α*, and ATF6. The application of 25 or 50 *μ*M Rg1 significantly decreased the expression level of these proteins (Figures 3(a)–3(d)). Known as critical mediator for attenuating ER stress, SIRT1 level was also examined in our study. In an agreement with previous findings, SIRT1 expression was depressed in A549 cells treated by LPS and was subsequently rescued by Rg1 treatment (Figures 3(e) and 3(f)). These data thus implicated that Rg1 suppressed ER stress at least partly through upregulating SIRT1 in A549 cells under *in vitro* mimicked septic conditions.

### 3.4. Rg1 Treatment Alleviated Lung Injury in Septic Mouse Model

**Figure 4:**
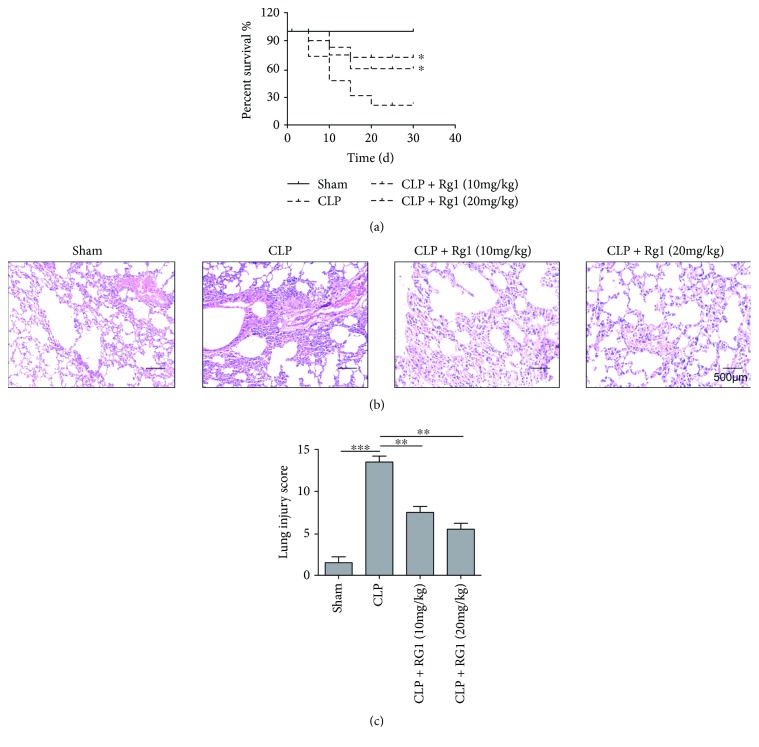
Rg1 treatment rescued septic-induced acute lung injury. (a) Survival rate of CLP mice treated with 10 or 20 mg/kg Rg1. (b) Representative lung tissue sections by HE staining. (c) Comparison of lung injury score. ^∗^*P* < 0.05; ^∗∗^*P* < 0.01; ^∗∗∗^*P* < 0.001, compared to the sham or CLP model group. Data were shown as mean + SD based on three independent experiments.

Having established the protective role of Rg1 against LPS-induced intracellular ER stress and cell death, we then validate this finding using the CLP mouse model to mimic sepsis *in vivo*. We treated CLP mice with 10 or 20 mg/kg Rg1 and extracted lung tissues for HE staining. Treatment of Rg1 remarkably improved mouse survival (Figure 4(a)). A primary glance of tissue pathology revealed that CLP mice presented severe leukocyte infiltration, tissue edema, hemorrhage, and thickening of alveoli compared to the sham group. The treatment of Rg1 remarkably alleviated tissue pathology development (Figure 4(b)). Appling a semiquantitative system for lung injury score, consistent results were found as Rg1 treatment remarkably decreased injury score which is increased by CLP induction (Figure 4(c)). These results illustrated the protective effect of Rg1 against sepsis-induced lung injury *in vivo*.

### 3.5. Rg1 Suppressed Inflammatory Response in CLP Mice

**Figure 5:**
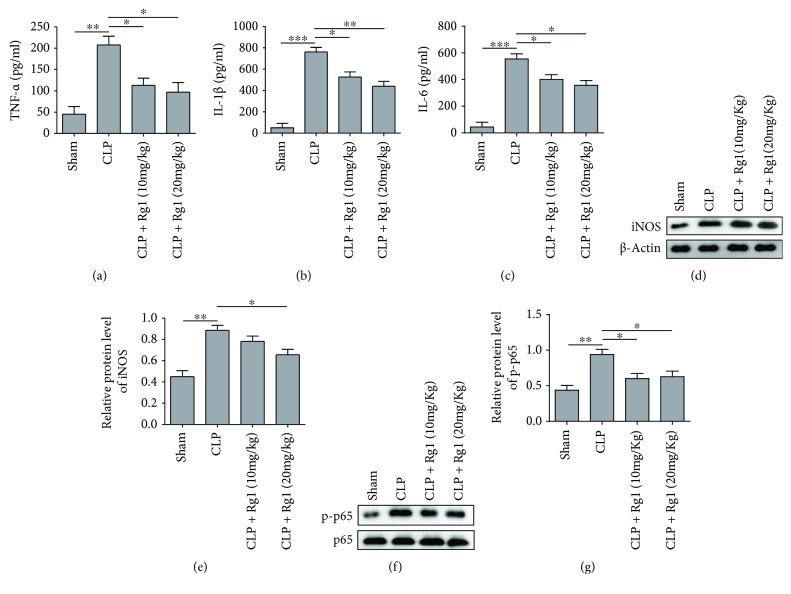
Rg1 inhibited inflammation response in CLP mice. (a–c) ELISA quantification for serum TNF-*α*, IL-1*β*, and IL-6 level in Rg1-treated CLP mice. (d) Representative images for iNOS proteins in lung tissue extracts by Western blotting. (e) Quantification of relative iNOS expression. (f) Representative images for p-p65 expression. (g) Quantification of relative expression level of p-p65. ^∗^*P* < 0.05; ^∗∗^*P* < 0.01; ^∗∗∗^*P* < 0.001, compared to the sham or CLP model group. Data were shown as mean + SD based on three independent experiments.

To substantiate the effect of Rg1 in protecting lung tissues from septic-related injury, we sought to investigate levels of inflammatory cytokines in general circulation of CLP mice. By ELISA quantification, we found that CLP mice had remarkably increased serum TNF-*α*, IL-1*β*, and IL-6 levels. Administration of 10 or 20 mg/kg Rg1 both significantly suppressed the release of those inflammatory cytokines (Figures 5(a)–5(c)). As further evidences support anti-inflammatory effects by Rg1, Western blotting in mouse lung tissues showed the inhibition of iNOS and p-p65 protein levels after Rg1 treatment (Figures 5(d)–5(g)). Therefore, CLP treatment elevated inflammation in mouse, and Rg1 treatment effectively alleviated inflammation, contributing to relieved lung tissue damage.

### 3.6. Rg1 Protected CLP Mice from Intracellular ER Stress

**Figure 6:**
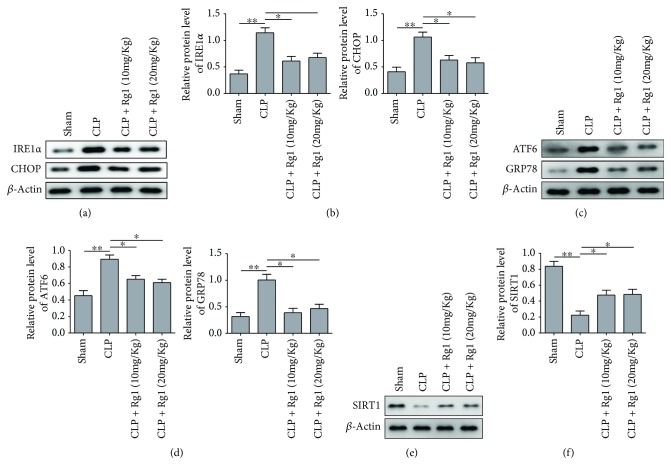
Rg1 treatment relieved ER stress in CLP mice. (a) Representative images for IER1*α* and CHOP proteins in lung tissue extracts from CLP mice by Western blotting. (b) Quantification of IER1*α* and CHOP protein levels. (c) Representative images ATF6 and GRP78 proteins by Western blotting. (d) Quantification of relative expression of ATF6 and GRP78. (e) Representative images for SIRT1 protein expression by Western blotting. (f) Quantification of relative expression of SIRT1. ^∗^*P* < 0.05; ^∗∗^*P* < 0.01, compared to the sham or CLP model group. Data were shown as mean + SD based on three independent experiments.

To confirm the results in A549 cell model, we lastly investigated whether Rg1 had similar effects in suppressing intracellular ER stress after sepsis induction. Western blotting showed that CLP mice presented a significantly elevated expression of ER stress marker proteins including CHOP, GRP78, IRE1*α*, and ATF6 compared to the sham control group. The expression of these proteins, however, was remarkably decreased after treating with 10 or 20 mg/kg of Rg1 in CLP mice (Figures 6(a)–6(d)). Lastly, we showed that CLP mouse had significantly lower SIRT1 expression, which was partially rescued after treating with Rg1 in a dosage-dependent manner (Figures 6(e) and 6(f)). In conclusion, Rg1 protected CLP mice from severe lung injury via SIRT1-mediated alleviation of intracellular ER stress and inflammation.

## 4. Discussion

In the present study, we described the relief of sepsis-induced acute pulmonary injury by ginsenoside Rg1, using both A549 cell culture and CLP mouse models. In clinics, systemic sepsis frequently leads to acute pulmonary injury, which can be caused by continuous exposure to circulating pathogen molecules such as endotoxin LPS that can trigger innate immune response [12]. It is estimated that over 40% sepsis patients develop acute pulmonary injury, which is characterized by inflammation and elevated vascular permeability [13]. The occurrence of lung injury sharply increases the overall mortality of sepsis patients and worsens the condition of septic shock [14]. However, no effective and specific treatment approach has been developed against inflammatory pulmonary injury secondary to sepsis. Therefore, the investigation for pathogenesis of sepsis-induced acute pulmonary damage is of critical importance for improving patient's prognosis.

ER stress is one crucial event for cytotoxicity and has been observed in sepsis, especially in those individuals with acute pulmonary injury [1]. ER stress can further induce the release of proinflammatory cytokines by activating downstream inflammatory pathways [15]. Such cascade activation of inflammatory further aggravates the programmed cell death and tissue injury. Ginsenoside Rg1 has been reported to have protective roles against various tissue injuries such as cerebral ischemic injury [16] and alcoholic hepatitis [17]. Many studies have identified the anti-inflammatory role of Rg1 in protecting against various neural damages [18]. In particular, Rg1 exerts potent anti-inflammatory response to protect lung tissues from LPS-induced injury by modulating macrophage infiltration [19]. However, no similar study has been reported regarding the role of Rg1 in protecting against sepsis-induced acute pulmonary injury. We thus provided the first piece of evidences showing that Rg1 could help to relieve lung tissues from ER stress and injury, as supported by a marked suppression of ER stress-related proteins, using both cultured cell model and CLP sepsis mouse model.

At the downstream of ER stress, inflammatory response and oxidative stress are two critical cellular events that lead to cell death. ER stress pathway is closely linked to cell inflammation, as TLR signaling activated by ER stress can strongly stimulate the production of proinflammatory cytokines [20]. As one critical mediator for ER stress, IRE1*α* has been shown to modulate cell apoptosis under ER stress conditions [21]. When examining oxidative stress inside cells, ER stress can induce oxidative stress inside cells, leading to further disease conditions including pulmonary fibrosis [22]. Here, we described both intracellular ER stress and oxidative stress levels. Results showed that Rg1 treatment remarkably suppressed cellular ER stress, inflammation, and oxidative stress, thus contributing to improved lung tissue conditions. We thus have reasons to believe that Rg1 effectively relieved ER stress, which further contributes to alleviated inflammation or ROS, as suggested by consistent results from cell culture or mouse models of sepsis.

SIRT1 has been reported in protecting against sepsis pathogenesis. For example, a recent study found that SIRT1 regulates inflammatory response of macrophages in sepsis, perhaps by mediating a long-coding RNA molecule [23]. In other disease model where SIRT1 has gained prominent improvement such as sepsis-induced liver injury in particular, such improving effects were dependent on SIRT1 pathway activation [24]. A more direct evidence found that SIRT1 directly attenuated sepsis-induced lung injury [25]. Here, we for the first time reported that Rg1 could exert its protective roles against sepsis\-related acute pulmonary injury. This study has certain limitations as only postinjury application of Rg1 was applied. It should have more clinical implications to examine if pretreatment using Rg1 could help to relieve or prevent sepsis-related lung injury. Moreover, whether Rg1 activated SIRT1 pathway by direct or indirect manner needs further investigation. In addition, we only used A549 carcinoma cell model to mimic *in vitro* conditions of sepsis. Although various studies used similar approach, potential bias may exist as A549 cells present altered radiosensitivity as the consequence of ER stress [26]. Therefore, further *in vitro* studies should be performed to reveal the involvement of Rg1-SIRT1 axis in alleviating ER stress.

In summary, our study found that ginsenoside Rg1 attenuated ER stress via upregulating SIRT1 to ameliorate sepsis-induced acute lung injuries both in LPS-induced pulmonary epithelial cells and CLP sepsis mouse models. Our finding suggested that Rg1 may be a potential candidate for the development of drug for patients with sepsis-induced inflammation and injury.

## Data Availability

All data generated or analyzed during this study are included within the article.
